# MATRIEX imaging: multiarea two-photon real-time in vivo explorer

**DOI:** 10.1038/s41377-019-0219-x

**Published:** 2019-11-28

**Authors:** Mengke Yang, Zhenqiao Zhou, Jianxiong Zhang, Shanshan Jia, Tong Li, Jiangheng Guan, Xiang Liao, Bing Leng, Jing Lyu, Kuan Zhang, Min Li, Yan Gong, Zhiming Zhu, Junan Yan, Yi Zhou, Jian K Liu, Zsuzsanna Varga, Arthur Konnerth, Yuguo Tang, Jinsong Gao, Xiaowei Chen, Hongbo Jia

**Affiliations:** 1 Key Laboratory of Optical System Advanced Manufacturing Technology, Changchun Institute of Optics, Fine Mechanics and Physics, Chinese Academy of Sciences, Changchun, 130033 China; 2 Graduate School, University of the Chinese Academy of Sciences, Beijing, 100039 China; 3 Brain Research Instrument Innovation Center, Suzhou Institute of Biomedical Engineering and Technology, Chinese Academy of Sciences, Suzhou, 215163 China; 4 Brain Research Center and State Key Laboratory of Trauma, Burns, and Combined Injury, Third Military Medical University, Chongqing, 400038 China; 5 Center for Neurointelligence, Chongqing University, Chongqing, 401331 China; 6 Center for Hypertension and Metabolic Diseases, Daping Hospital, Chongqing, 400042 China; 7 Advanced Institute of Brain and Intelligence, Guangxi University, Nanning, 530005 China; 8 Centre for Systems Neuroscience, Department of Neuroscience, Psychology and Behaviour, University of Leicester, Leicester, UK; 9 Institute of Neuroscience, Technical University Munich, 80802 Munich, Germany

**Keywords:** Multiphoton microscopy, Ca2+ imaging

## Abstract

Two-photon laser scanning microscopy has been extensively applied to study in vivo neuronal activity at cellular and subcellular resolutions in mammalian brains. However, the extent of such studies is typically confined to a single functional region of the brain. Here, we demonstrate a novel technique, termed the multiarea two-photon real-time in vivo explorer (MATRIEX), that allows the user to target multiple functional brain regions distributed within a zone of up to 12 mm in diameter, each with a field of view (FOV) of ~200 μm in diameter, thus performing two-photon Ca^2+^ imaging with single-cell resolution in all of the regions simultaneously. For example, we demonstrate real-time functional imaging of single-neuron activities in the primary visual cortex, primary motor cortex and hippocampal CA1 region of mice in both anesthetized and awake states. A unique advantage of the MATRIEX technique is the configuration of multiple microscopic FOVs that are distributed in three-dimensional space over macroscopic distances (>1 mm) both laterally and axially but that are imaged by a single conventional laser scanning device. In particular, the MATRIEX technique can be effectively implemented as an add-on optical module for an existing conventional single-beam-scanning two-photon microscope without requiring any modification to the microscope itself. Thus, the MATRIEX technique can be readily applied to substantially facilitate the exploration of multiarea neuronal activity in vivo for studies of brain-wide neural circuit function with single-cell resolution.

## Brain imaging: Seeing neurons in action in multiple brain regions simultaneously

The two-photon scanning microscopy imaging technique, commonly applied to study neuronal activity in single regions of mammalian brains, has now been adapted to observe differently functioning regions simultaneously. The innovation, called multiarea two-photon real-time in vivo explorer (MATRIEX), has been developed by Hongbo Jia and colleagues at the Suzhou Institute of Biomedical Engineering and Technology, Chinese Academy of Sciences, with co-workers in other institutions in China and Europe. Any conventional two-photon microscope can be transformed into a MATRIEX microscope by attaching an additional component. The different regions examined simultaneously, each around 200 μm in diameter, can be within a study zone of up to 12 mm in diameter and at depth intervals of more than 1 mm. The technique has been demonstrated by visualizing the transformation of both intraregional and interregional neuronal pairwise activity correlations from the anesthetized state to the awake state in the primary visual cortex, the primary motor cortex and the hippocampal CA1 region of mice. It will facilitate understanding of the functions of three-dimensional brain-wide neural circuits at single-cell resolution in vivo.

## Introduction

Two-photon laser scanning microscopy, originally developed in the 1990s^1^, has been widely applied and is particularly popular among neuroscientists interested in studying neural structures and functions in vivo^2^. A major advantage of two-photon (and three-photon^3^) imaging technology for the study of living brains is the optical resolution that is achieved in densely labeled brain tissues that strongly scatter light^4^, in which optically sectioned image pixels are scanned and acquired with minimal crosstalk. Unfortunately, this advantage also induces a major drawback; i.e., simultaneously viewing objects (with an update rate of at least 5 Hz) is highly restricted within a certain range of the lateral and axial distances, which is typically <1 mm. Many techniques have been successfully established to extend such limits. For example, the axial limit can be extended by scanning through multiple focal planes at different focal depths^5,6^ or by using Bessel-shaped laser beams with prolonged focal point-spread functions to image all planes within a certain depth range^7^; the lateral limit can be extended by using large-field low-magnification objectives^8^ with multiplexed subfield scanning techniques^9–12^ or by using fast mechanical rotating devices to rapidly switch between different fields of view (FOVs)^13^. However, two major issues have limited the effectiveness of these techniques. First, one technique alone can extend the limit to >1 mm in either the lateral or the axial dimension, but not both. Second, these techniques typically rely on highly customized electronic devices and optical components that are not commercially available. Consequently, it has been difficult to implement such techniques in neuroscience research laboratories.

Nonetheless, there is an increasingly high demand in neuroscience to investigate brain-wide neuronal functions in vivo with single-cell resolution. For example, cognitive tasks such as visual-cue-guided navigation^14,15^ involve neural circuits consisting of multiple functional regions, including the cortex and the hippocampus. To address fundamental questions of how individual neuronal activities in a brain-wide neural circuit are involved in perception, cognition and behavior, it is far from sufficient to observe and/or manipulate single-neuron activities in only one brain region. Considering the possible number of brain region combinations that potentially need to be studied, the future need for multiregion imaging is expected to be much higher than the need for conventional single-region imaging. A straightforward approach is simply to place two microscopes (independent scanning-acquisition systems) above the same animal brain^16^. A recent report successfully demonstrated that by using such an approach, the cortex and the cerebellum can be simultaneously imaged^16,17^. However, imaging more than two brain regions with this approach leads to substantial increases in cost and complexity.

Together, these high expectations for both performance and feasibility pose a highly challenging engineering question: how can a single imaging system (with one scanning-acquisition device) obtain live microscopic images (with single-cell resolution at a frame rate of at least 5 Hz) simultaneously from multiple in vivo brain regions that are distributed across large lateral and axial distances (>1 mm)? To address this question, we introduce a new method that combines two-stage magnification and multiaxis optical coupling. This method is realized by using a low-magnification dry objective (DO) with multiple water-immersed miniaturized objectives (MOs) under the DO. Configuring MOs with different lengths can result in multiple object planes that are distributed at different depths. With the help of standard mechanical micromanipulation devices under the DO and the ability to observe tissue through either the conventional microscope oculars or the two-photon imaging system, each of the MOs can be placed at the desired target position and depth in the brain tissue. Thus, the new compound objective assembly can be used in almost the same way as the original water-immersed microscope objective and does not require any modification to the image scanning and acquisition subsystems of the microscope.

## Results

### The MATRIEX compound objective assembly

**Fig. 1:**
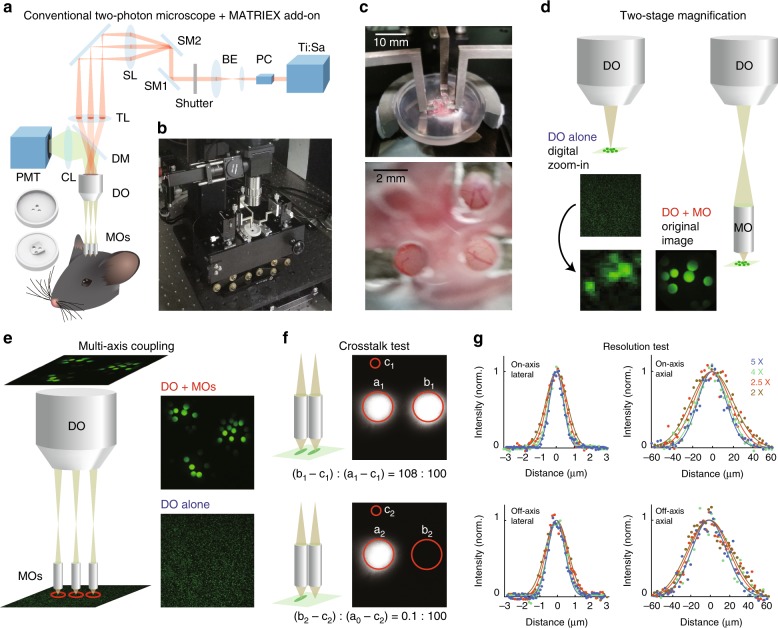
Design and implementation of MATRIEX imaging. **a** Experimental diagram of the MATRIEX imaging system. The two round 3D objects in the lower-left corner are the top and bottom views of the mouse head chamber used for in vivo imaging. (Ti:Sa): Ti:Sapphire ultrafast pulsing laser; PC: Pockels cell; BE: beam expander; SM1 and SM2: *x*–*y* scanning mirrors; SL: scan lens; TL: tube lens; DM: dichroic mirror; CL: collection lens; PMT: photomultiplier tube; DO: dry objective; MOs: miniaturized objectives. **b** Photograph showing an oblique overview of the actual MATRIEX imaging system. **c** The photograph in the upper image shows a zoomed in view of the three MOs attached to the manipulating bars over the head chamber; the lower photograph was taken directly above the MOs with a smartphone camera. All MOs used in this figure are of the same model: ‘standard version’ (see Table 1). **d**, **e** Illustrations of the two-stage magnification and multiaxis coupling. The square images are actual two-photon images taken of 20-μm beads. Each red circle indicates one FOV. The model of DO used in panels d-f is the Olympus MPlan ×4/0.1, and all MOs in this figure are of the same customized model (‘Standard version’, see Table 1). **f** Illustration showing the absence of inter-FOV crosstalk under adjacent MOs. The images were taken on a uniform fluorescent plate. The red circles indicate the areas of analysis used to compare the image contrast between two conditions; the left-side condition shows the fluorescent plate under both MOs, and the right-side condition shows the fluorescence plate under only one MO. **g** Testing the optical resolution of the compound assembly with 0.51-μm beads. Curves: Gaussian fittings of raw data points. The on-axis or off-axis fluorescence intensity profiles were measured when the axis of the MO was aligned with the axis of the DO or apart from the axis of the DO (2 mm for the DO of ×4 or ×5, 3 mm for the DO of ×2.5, and 4 mm for the DO of ×2), respectively. See Table 2 for a summary of the measurements.

Figure 1a shows the basic concept of the MATRIEX technique. In a two-photon laser scanning microscope equipped with a conventional single-beam raster scanning device, the conventional water-immersion microscope objective with a medium or high magnification (typically, ×16–×40) is replaced by a customized compound objective assembly (Fig. 1a; see the three small cylinders and one large cylinder above the head of the mouse). The compound assembly consists of multiple MOs, each with a lateral magnification factor of ~×8 (gradient-index (GRIN) lens; see Table 1 for detailed specifications; this type of lens is broadly used for miniaturized microscopes^18^), and a low-magnification DO (×2–×5 magnification; a broad range of low-cost standard industrial products are available). The MOs are inserted through multiple craniotomies, during which a 3D-printed plastic chamber glued to the skull roughly aligns the MOs with some space to adjust both the lateral position and depth. The precise manipulation of the individual MOs is performed by individually moving each metal bar that attaches an MO at the tip (Fig. 1b, c). When each MO is placed at the proper focal depth, the objects under all MOs can be visualized simultaneously in the same image plane (for example, see the microvascular patterns in three FOVs acquired by a simple smartphone camera in the lower image of Fig. 1c).

**Table 1:** Specifications of the GRIN lenses used in this study.

	Standard version (used for the cortex)	Long-coupling version (used for the hippocampus)
Refractive index of the material on the central axis *N*_0_	1.643	1.643
Pitch (*P*)	0.261	0.259
Distribution coefficient of the refractive index $\left( {\sqrt A } \right)$	0.299	0.311
Length $\left( {Z = 2\pi P/\sqrt A } \right)$	5.49 mm	5.236 mm
Front working distance (*L*_1_)	0.15 mm	0.15 mm
Refraction index of the front working medium (water, *n*_1_)	1.328	1.328
Refraction index of the back working medium (air, *n*_2_)	1.000	1.000
Back working distance $\left( {L_2 = \frac{{ - (n_1n_2/\sqrt A ){\mathrm{sin}}(Z\sqrt A ) - n_2N_0L_1{\mathrm{cos}}(Z\sqrt A )}}{{n_1N_0\cos \left( {Z\sqrt A } \right) - N_0^2L_1\sqrt A {\mathrm{sin}}(Z\sqrt A )}}} \right)$	16 mm	17.3 mm

The parameters followed by equations are calculated with other parameters

The MATRIEX technique is based on two principles: two-stage magnification and multiaxis coupling. Figure 1d illustrates two-stage magnification, in which 20-μm beads appear as tiny and blurry dots when viewed through the DO alone but as crisp round circles when viewed through the compound assembly. Figure 1e illustrates multiaxis coupling, in which a single DO is coupled with multiple MOs along different axes. Multiple object planes under the MOs are all conjugated on the same image plane. Note that multiple object planes can be in the same or different geometrical planes, depending on whether the MOs have the same or different parameters (Fig. 1e shows the configuration when using the same MOs). A simple raster scan in a rectangular frame results in the acquisition of a rectangular image including multiple circular FOVs, with each FOV corresponding to one MO. The sequential pixel scanning principle of two-photon microscopy ensures that the inter-FOV pixel crosstalk is minimal. To illustrate this point, we measured the inter-FOV pixel crosstalk by comparing the average brightness of pixels in the two FOVs corresponding to two tightly adjacent MOs (Fig. 1f). When fluorescent objects of similar brightness (homogeneous fluorescent plates) are under both MOs, the average pixel values in both FOVs are similar (108: 100); when there is no fluorescent plate under one MO, the pixel value in the corresponding FOV is just 0.1% of the brightness of the FOV with a fluorescent plate. Thus, inter-FOV pixel crosstalk is not a concern in practice.

### Optical resolution of the compound assembly

The mechanism that allows the compound assembly to achieve a better resolution than the resolution of the DO alone is the numerical aperture (NA) magnification: the NA of the excitation light cone under the DO is magnified by the angular magnification factor of the MO. The NA of the compound assembly, NA_comp_ is given by the following equation:

1 $${\mathrm{NA}}_{{\mathrm{comp}}} = n_1{\it{{\mathrm{sin}}}}(\theta _1) \approx n_1{\mathrm{sin}}(\theta _0)/M_{\mathrm{a}} = \left( {\frac{{1.33}}{{M_{\mathrm{a}}}}} \right){\mathrm{NA}}_{{\mathrm{DO}}}$$

where *n*_1_ = 1.33 is the refractive index of water, NA_DO_ is the nominal NA value of the DO, and *M*_a_ = *θ*_0_/*θ*_1_ = 0.1675 is the angular magnification factor of the MO (given by the manufacturer). However, note that the ‘effective NA’ for the excitation light cone must not exceed the nominal NA value of the MO (NA_MO_). Thus, the ‘effective NA’ of the excitation light cone at the biological specimen, NA_eff_, is given by the following equation:

2 $${\mathrm{NA}}_{{\mathrm{eff}}} = \min \left( {{\mathrm{NA}}_{{\mathrm{comp}}},\,{\mathrm{NA}}_{{\mathrm{MO}}}} \right)$$

In practice, we have chosen GRIN lenses as the MOs. GRIN lenses can be very flexibly custom-designed and easily mass-produced at low cost, which greatly facilitates the experimental design. The employed MOs have a nominal NA value of ~0.5 (0.483 as given by the manufacturer). Thus, Eqs. (1) and (2) show that when using a DO with a very low NA, such as the Mitutoyo ×2 (NA = 0.055), the calculated NA_comp_ (= 0.44) is the ‘effective NA’, NA_eff_, whereas when using DOs with a higher NA (Olympus ×2.5/0.08, ×4/0.1, and ×5/0.1), NA_eff_ is capped and equal to NA_MO_ (0.483). Note that these calculations are dependent upon the axis of the MO being aligned with the axis of the DO. In practice, for multiaxis coupling, the axis of each MO is typically misaligned from the axis of the DO (‘off-axis’). Thus, we measured the lateral and axial resolutions using fluorescent beads for both the ‘on-axis’ (MO axis aligned with the DO axis) and ‘off-axis’ configurations (Fig. 1g and Table 2). The results showed that the ‘off-axis’ resolutions are rather comparable to the ‘on-axis’ resolutions.

**Table 2:** NA values and measured optical resolutions for compound assemblies using DOs of different models (Fig. 1g) and the ‘standard version’ MO (Table 1).

DO magnification and model	×5 Olympus MPlan N	×4 Olympus MPlan N	×2.5 Olympus MPlan FL N	×2 Mitutoyo MPlan Apo
Diameter of the target zone	4.8 mm	6 mm	9.6 mm	12 mm
NA_DO_ given by the manufacturer	0.1	0.1	0.08	0.055
NA_eff_ of the compound assembly	0.48	0.48	0.48	0.43
On-axis lateral resolution	1.0 ± 0.1 μm	1.1 ± 0.2 μm	1.4 ± 0.1 μm	1.5 ± 0.1 μm
On-axis axial resolution	35 ± 1 μm	37 ± 3 μm	42 ± 2 μm	51 ± 3 μm
Distance between MO and DO axes for off-axis measurements	2 mm	2 mm	3 mm	4 mm
Off-axis lateral resolution	1.3 ± 0.1 μm	1.4 ± 0.3 μm	1.6 ± 0.2 μm	1.8 ± 0.2 μm
Off-axis axial resolution	46 ± 2 μm	45 ± 2 μm	48 ± 1 μm	54 ± 3 μm

The resolutions are expressed by the full width at half maximum (FWHM) of 0.51-μm fluorescent beads (Bangs Laboratories, FC03F) measured (mean ± s.e.m.) from the acquired images. The image scale for each different configuration is individually calibrated using 20-μm fluorescent beads (Sicastar-greenF, 42-00-204)

### Enabling multiple FOVs at large axial and lateral distances

**Fig. 2:**
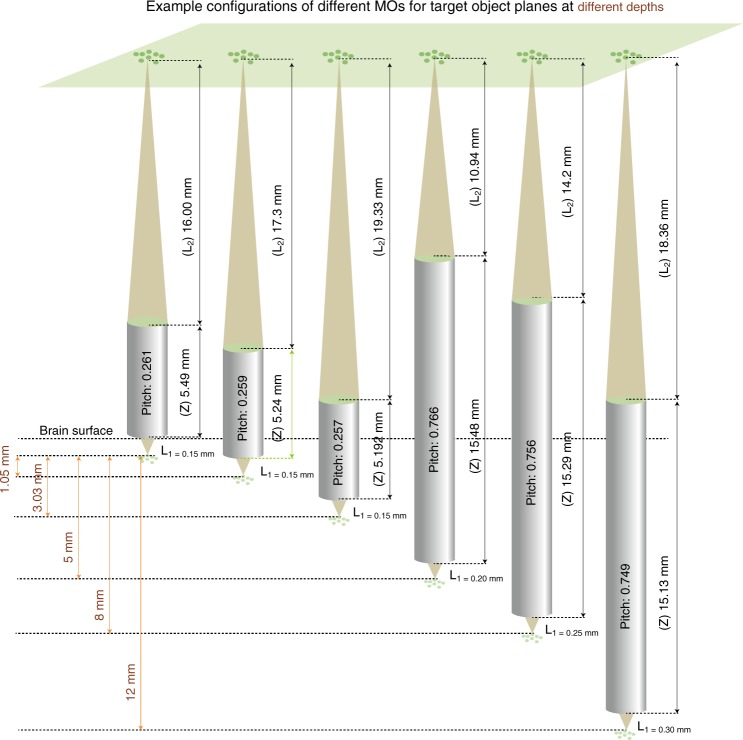
Configuring the MOs (GRIN lenses) with different parameters to target object planes at different depths to then be conjugated on the same image plane. Each gray cylinder represents one lens with a pitch value, front working distance (*L*_1_), back working distance (*L*_2_) and length (*Z*).

The key advantage of the MATRIEX technique is the ability to simultaneously acquire images of multiple objects at large depth intervals, e.g., >1 mm. To illustrate this point, we designed different types of MOs with different parameters. When each of the different MOs is placed at a specified depth, the corresponding object planes can be conjugated with the same image plane (Fig. 2). However, in practice, there are two constraints for the calculation of lens parameters to achieve the desired target object plane depths. First, both the back working distance (*L*_2_) and the length (*Z*) vary with the pitch value (*P*) of the GRIN lens (see Table 1 for details). Second, the lens must have a portion outside the brain tissue or guide cannula to be positioned and fixed. Considering these two constraints, we provide a reference graph for the pitch value and the other corresponding parameters with respect to the target imaging depth (relative to the cortical surface), as shown in Fig. 2. In practical experiments, minor mismatches between the desired object plane depth and the actual object depth can be well compensated by adjusting the MOs individually along each of the *z*-axes.

**Fig. 3:**
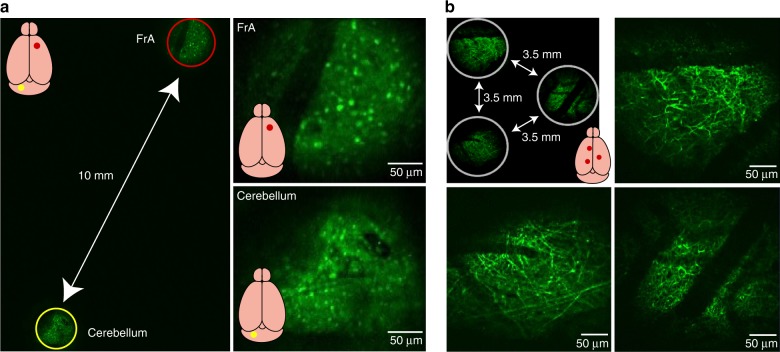
Demonstration of MATRIEX imaging: structural imaging in multiple brain areas in vivo. **a** Left image: a full-frame image including two FOVs in the frontal association cortex (FrA) and the cerebellum. The red and yellow circles indicate two FOVs that are digitally enlarged and shown in the upper-right and lower-right images. A GAD67-GFP transgenic mouse (with the interneurons labeled brain-wide) was used. Two MOs (‘standard version’) were placed at the same depth under a DO (Mitutoyo ×2/0.055). **b** Example configuration of three FOVs in the cortex of a Thy1-GFP transgenic mouse (with layer 5 cortical neurons specifically labeled and with tuft dendrites visible near the cortical surface). Three MOs (‘standard version’) were placed at the same depth under a DO (Olympus ×4/0.1).

The maximum lateral size of the targetable zone is limited by the maximum size of the scanning field under the DO of the conventional microscope. Typically, for a DO with a ×2 magnification, the diameter of the targetable zone is ~12 mm, which is nearly the size of an entire adult mouse brain. For example, as shown in Fig. 3a, the frontal association cortex and the cerebellum of the mouse can be simultaneously imaged. However, in practice, using a ×4 air objective as the DO is also suitable for many target region configurations (within a zone of ~6 mm in diameter) and achieves a better resolution than that for a DO with a ×2 magnification (Fig. 1g). For example, three cortical areas in a triangular configuration (with each pair separated by a distance of 3.5 mm) can be imaged with a compound assembly using a ×4 DO, with which fine dendritic structures are readily visible, as shown in Fig. 3b.

### Simultaneous imaging in V1, M1, and CA1 of anesthetized and awake mice

**Fig. 4:**
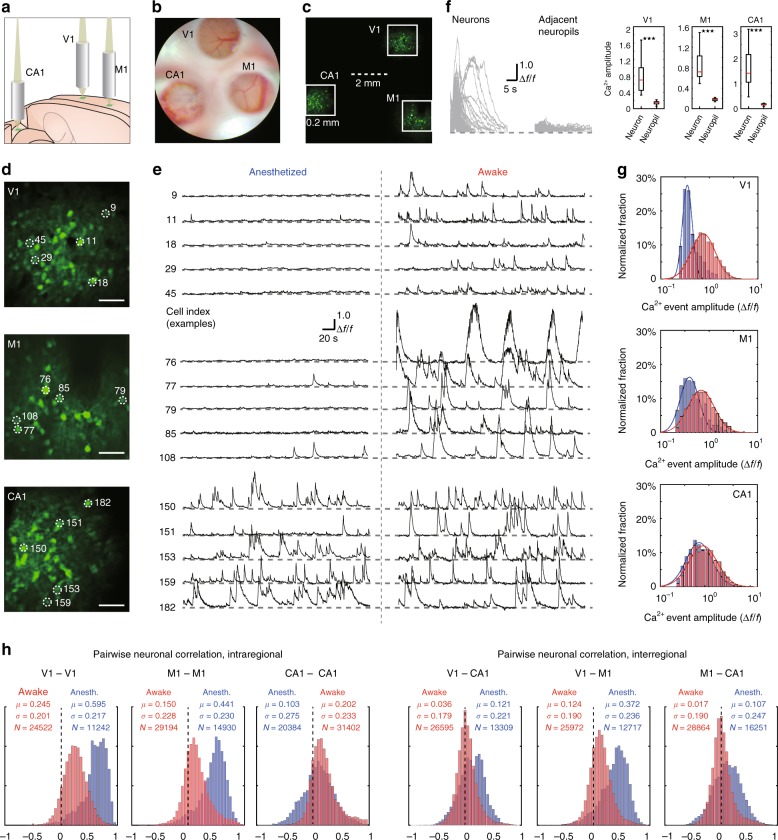
Demonstration of MATRIEX imaging: simultaneously acquiring live neuronal activity patterns in V1, M1, and hippocampal CA1 in mice in the anesthetized state or awake state. The neurons were labeled by a genetically encoded fluorescent Ca^2+^ indicator, GCaMP6f (see Supplementary Materials for details). **a** Illustration showing the positioning of three MOs over the V1, M1 and hippocampal CA1 regions in a model mouse brain. **b** A camera photograph taken through the microscope ocular lens under white light bright-field illumination, in which three FOVs are readily visible. The upper region is V1, the lower-left region is CA1, and the lower-right region is M1. **c** A two-photon image, which is an average of 100 frames, acquired by simple full-frame raster scanning with a two-photon microscope. The solid white boxes show the three parts of the image that are enlarged in panel (**d**). **d** Digitally enlarged individual FOVs showing neurons in V1, M1, and CA1, from top to bottom. Scale bar: 40 μm. **e** Time-lapse Ca^2+^ signal traces of five example cells from each region, with each labeled by the cell index. Recordings of the same cell in the same animal in the anesthetized state (left side) and in the awake state (right side) are shown. **f** Left: traces showing individual Ca^2+^ signal events (split from each onset time and overlaid) from randomly selected example cells. Middle: Ca^2+^ signal traces of each of the neuropil zones that are directly adjacent to each of the example cells. Right: three box plots comparing the neuronal Ca^2+^ signal event amplitude to the neuron’s adjacent neuropil Ca^2+^ signal amplitude; paired Wilcoxon rank sum test, ***P < 0.001. **g** Log-normal fitting of the distribution histograms of the spontaneous Ca^2+^ event amplitude for data pooled from all animals. The red bars and fitted curve show the distribution of data recorded in the awake state, and the blue bars and fitted curve show the distribution of data recorded in the anesthetized state. **h** Pairwise neuronal activity correlation (Pearson correlation coefficients) for data pooled from all animals. The red bars show the distribution of data recorded in the awake state, and the blue bars show the distribution of data recorded in the anesthetized state.

As a practical example of the application of the MATRIEX technique, we performed simultaneous two-photon Ca^2+^ imaging of GCaMP6f-labeled neurons in the primary visual cortex (V1), primary motor cortex (M1) and hippocampal CA1 region of mice. The V1 and M1 regions were imaged ipsilaterally (left hemisphere), and the CA1 region was imaged contralateral to V1 and M1. The illustration in Fig. 4a shows the configuration of the three MOs, in which the two MOs for V1 and M1 were placed directly above the cortex, and the MO for the hippocampal CA1 region was inserted after the surgical removal of the cortical tissue above. The surgical insertion procedure was similar to previously described procedures^19,20^. For V1 and M1, we used the ‘standard version’ lens, and for CA1, we used the ‘long-coupling version’ lens (see Table 1 for details). The design of these lenses ensured that object planes corresponding to V1, M1, and CA1 could be conjugated on the same image plane (Fig. 4b). Using a conventional two-photon microscope equipped with a 12 kHz resonant scanner, we scanned the full image frame with 1200 × 1200 pixels at 10 Hz (unidirectional scan). In the full scanning frame, the three FOVs were readily visible (Fig. 4c), and we enlarged three different parts of the full image to show single cells (Fig. 4d). Note that there were two scales in one frame of the image, i.e., the interregional scale (as shown by the dashed scale bar in Fig. 4c; 2 mm) and the intraregional scale (as shown by the size of the solid box in Fig. 4c; 0.2 mm). Thus, instead of using one entire image (1200 × 1200 pixels) to map the entire imaging zone (~4.3 mm × 4.3 mm in size, reduced from 6 mm × 6 mm by a scanner zoom factor of ×1.4) with the pixel size being too coarse (~4 μm), three parts of the image (~320 × 320 pixels each) were used to map the three FOVs (~0.2 mm × 0.2 mm each) with the pixel size (~0.6 μm) matching the Nyquist sampling rate for an optical resolution of 1.0−1.4 μm and, thus, being sufficient to resolve single neurons. Note that this result was obtained primarily by the optical design and not by the laser scanning control or image acquisition settings because the laser scanning and image acquisition systems were configured in exactly the same way as in conventional single-FOV two-photon microscopy. We measured the laser power by placing a standard laser power meter underneath the MOs with the same DO–MO configuration and imaging parameters as those used in the in vivo experiments. We filled the space between and around the MOs with black paper such that the power meter sensor was exposed only to light through the DO–MO assembly, and the measured power was 112 mW. However, note that the laser power was distributed among multiple FOVs (e.g., for the 3-MO configuration, in each FOV the laser power was ~37 mW).

We recorded spontaneous activity continuously for 300 s; see Supplementary Video 1 for an example in one animal. For this animal, we show the Ca^2+^ signal traces of the same example neurons (selected from each brain region) first in the anesthetized state and, then, in the awake state (Fig. 4e). We measured the Ca^2+^ signals in neuropil regions adjacent to neuronal cell bodies and found that the neuropil signals were largely negligible in all three brain regions (Fig. 4f): V1 (Δ*f*/*f* value, neurons: 0.7\0.4–1.0, neuropils: 0.15\0.11–0.17, *N* = 41, paired Wilcoxon rank sum test, *P* = 6e−15), M1 (Δ*f*/*f* value, neurons: 0.7\0.6–1.0, neuropils: 0.18\0.15–0.19, *N* = 12, paired Wilcoxon rank sum test, *P* = 3e−5), and CA1 (Δ*f*/*f* value, neurons: 1.3\1.0–2.1, neuropils: 0.17\0.14–0.19, *N* = 41, paired Wilcoxon rank sum test, *P* = 6e−15). A complete cell-by-cell demonstration of the Ca^2+^ signals in both anesthetized and awake states is shown in Suppl. Fig. 1, in which the signal quality of each single cell and the dynamic range across different cells can be readily observed. We pooled the single-cell analysis results from six recordings in the anesthetized state and eight recordings in the awake state from different animals (two recordings were made in the same animals in both states, whereas the rest of the recordings were made in different animals in one state each). Interestingly, all of the histograms of the Ca^2+^ event amplitudes (in each region and for each state) can be fitted by highly skewed, log-normal distributions^21^, as shown in Fig. 4g. In terms of the log-scaled distributions of the Ca^2+^ event amplitudes, the V1 and M1 neurons underwent drastic upshifts from the anesthetized state to the awake state (V1, anesthetized: *μ* = 10^−0.46^, *σ* *=* 10^0.14^, *N* = 125, awake: *μ* = 10^−0.13^, *σ* = 10^0.35^, *N* = 430, Wilcoxon rank sum test, *P* = 4e−74; M1, anesthetized: *μ* = 10^−0.30^, *σ* = 10^0.31^, *N* = 211, awake: *μ* = 10^−0.03^, *σ* *=* 10^0.44^, *N* = 483, Wilcoxon rank sum test, *P* = 4e−63), while CA1 neurons underwent a minor upshift that was also highly significant (anesthetized: *μ* *=* 10^−0.13^, *σ* = 10^0.41^, *N* = 342; awake: *μ* = 10^−0.04^, *σ* = 10^0.45^, *N* = 493, Wilcoxon rank sum test, *P* = 4e−25).

The abovementioned results could have been obtained by alternative techniques by sequentially performing conventional single-FOV imaging in one brain region after another. However, the MATRIEX technique provided data with extra dimensions of information that single-FOV imaging techniques cannot offer, e.g., both interregional and intraregional neuronal pairwise correlations in real-time. In this set of experiments, there were three pairs of interregional correlations (V1–M1, V1–CA1, and M1–CA1) and three pairs of intraregional correlations (V1–V1, M1–M1, and CA1–CA1); the histograms of each correlation are shown in Fig. 4h. Interestingly, all of the correlations except for the CA1–CA1 correlation underwent substantial downshifts from the anesthetized state to the awake state (see the statistical parameters in Fig. 4h). In contrast, the CA1–CA1 correlation underwent an upshift from the anesthetized state to the awake state. Another interesting finding was that for both the anesthetized state and awake state, the cortico-hippocampal interregional correlations (V1–CA1 and M1–CA1) were both much smaller than the cortico-cortical interregional correlation (V1–M1). Statistical tests for each of the above statements regarding the pairwise neuronal correlation levels yielded significant results with the *P* value being 0 (too small to be calculated). Taken together, these results show a highly inhomogeneous distribution and transformation of spontaneous activity patterns from the anesthetized state to the awake state at the brain-wide circuit level with single-cell resolution. In another example experiment using the same configurations of MO, DO, and target imaging regions, we show visual stimulation-related neuronal activities in V1, M1, and CA1 in awake mice (Suppl. Fig. 2).

## Discussion

The MATRIEX technique, based on the principle of two-stage magnification and multiaxis optical coupling, has enabled the simultaneous two-photon Ca^2+^ imaging of neuronal population activities in multiple brain regions at different depths (e.g., V1, M1, and CA1) with single-cell resolution in anesthetized and awake mice. The uniqueness of the MATRIEX imaging method is its ability to simultaneously image multiple brain areas that are distributed at very different coordinates, both axially and laterally (more than 1 mm apart, up to 12 mm), which is nearly impossible to achieve with conventional two-photon microscope systems based on a single optical axis. Strikingly, any conventional two-photon microscope can be transformed into a MATRIEX microscope, and all of the original microscope functionalities are preserved. The key to enabling such a transformation is the design of a compound objective assembly as a flexible add-on module. In particular, note that conventional two-photon laser scanning systems are fully compatible with the compound assembly (because the DOs are industrial products with the same conventional standards) and do not need to be modified. In contrast, other novel custom-made objectives for imaging in a single large FOV^9,10^ require highly customized laser scanning devices that are barely compatible with the majority of the currently available two-photon microscopes worldwide (that are built by conventional standards). Placing two independent sets of two-photon laser scanning rigs over one animal head can also realize dual-region imaging^16,17^, however, due to practical limitations related to the size of the scanning rigs (but not the multiple GRIN lenses that are directly attached to the skull), configurations of three or more brain regions are very difficult to realize geometrically using such techniques. See Suppl. Table 1 for a survey of the currently available multiregion imaging techniques applied in neuroscience.

We must mention one drawback of using GRIN lenses as MOs: GRIN lenses significantly lack certain optical aberration correction properties. As a result, the lateral resolution (measured by fluorescent beads) is good, but the measured axial resolution is relatively poor (Fig. 1g). Indeed, there are MOs with higher optical quality (and with higher cost and less flexibility), e.g., a special MO made by combining multiple pieces of precision-machined high-quality optical glass^22^. However, the MOs are usually single-use consumables, particularly when the MOs are glued to the skull of the animal for chronic imaging experiments. Thus, after considering the trade-offs, we decided to sacrifice some axial resolution to improve the feasibility of the practical experiments.

By using different carefully designed MOs to suit different brain regions, the MATRIEX technique essentially offers a high-flexibility ‘three-dimensional multiarea optical interface’ between neural network activity in the animal brain and a two-photon laser scanning system. As shown above, there is 100% compatibility between conventional two-photon microscopy systems and the MATRIEX technique. Therefore, a broad range of other advanced techniques developed for two-photon microscopy are also compatible and may be combined with the MATRIEX technique to further extend the performance and applicability, including, but not limited to, (1) random-access scanning and/or photostimulation methods^23–26^; (2) advanced surgical techniques for chronic imaging^27^; and (3) adaptive optics^28^ and other point-spread-function shaping techniques^29^. Overall, we expect that the application of the MATRIEX technique will substantially advance the study of three-dimensional brain-wide neural circuit dynamics with single-cell resolution.

## Materials and methods

In this study, we used a “LotosScan” two-photon microscope (Suzhou Institute of Biomedical Engineering and Technology, Chinese Academy of Sciences). This microscope is based on conventional single-beam resonant scanning technology, similar to our prototypes reported or used in earlier studies^22,30–35^ and similar to several other commercially available products. The implementation of the MATRIEX technique does not require the two-photon microscope to be custom-built, i.e., any two-photon microscope that uses a commercial standard objective can be transformed into a MATRIEX microscope. A detailed description of the components of the microscope that we used in this study is given in the Supplementary Materials.

Adult male C57BL/6J mice (8–10 weeks old) were used in this study. All animals were provided by the Laboratory Animal Center at the Third Military Medical University. All surgical tools and optical parts that were in contact with the animals were sterilized before use. All the experimental procedures were performed in accordance with institutional animal welfare guidelines, were approved by the Third Military Medical University Animal Care and Use Committee and were similar to the procedures used in our earlier reports^36–39^. A detailed description of the surgical procedure and fluorescence labeling is given in the Supplementary Materials.

We analyzed our data using custom-written software in LabVIEW 2012 (National Instruments), Igor Pro 5.0 (Wavemetrics), and MATLAB 2014a (MathWorks). To correct motion-associated artifacts in imaging data^40^, we used a frame-by-frame alignment algorithm to minimize the sum of squared intensity differences between each frame image and a template generated by averaging the selected image frames. To extract fluorescence signals, we visually identified neurons and drew regions of interest (ROIs) based on fluorescence intensity. Fluorescence changes (*f*) were calculated by averaging the corresponding pixel values in each specified ROI. Ca^2+^ signals were expressed as relative fluorescence changes Δ*f*/*f*  = (*f* − *f*_0_)/*f*_0_, where the baseline fluorescence *f*_0_ was estimated as the 25th percentile of the entire fluorescence recording. We used the Pearson correlation coefficient to calculate the pairwise neuronal correlation. Let *X* and *Y* be the arrays of data points in the Ca^2+^ signal traces of neurons X and Y, respectively. The Pearson correlation coefficient was calculated as follows: ${\mathrm{corrcoef}}(X,Y) = \frac{{{\mathrm{cov}}(X,Y)}}{{\sigma _X \cdot \sigma _Y}}$, where cov(*X*,*Y*) represented the covariance between *X* and *Y*. *σ*_*X*_ is the standard deviation of *X*. All biological measurement results were expressed in the format of ‘median\25th–75th percentiles’, and the Wilcoxon rank sum test was applied for all statistical tests, if not stated otherwise.

## Supplementary information

Supplementary Materials

Supplementary Video 1
