# Supplementary material for: MATRIEX imaging: multiarea two-photon real-time in vivo explorer
Source: Light Sci Appl. 2019 Nov 28;8:109. doi: 10.1038/s41377-019-0219-x (PMC6881438; doi:10.1038/s41377-019-0219-x)
Supplement: Supplementary file 1 — Supplementary Materials [file 41377_2019_219_MOESM1_ESM.docx]

**MATRIEX Imaging: Multiarea Two-photon Real-time In vivo Explorer**

**Authors**

Mengke Yang^1,2,3^, Zhenqiao Zhou^3^, Jianxiong Zhang^4^, Shanshan Jia^4^, Tong Li^4^, Jiangheng Guan^4^, Xiang Liao^5^, Bing Leng^3^, Jing Lyu^3^, Kuan Zhang^4^, Min Li^3^, Yan Gong^3^, Zhiming Zhu^6^, Junan Yan^7^, Yi Zhou^7^, Jian K Liu^8^, Zsuzsanna Varga^9^, Arthur Konnerth^9^, Yuguo Tang^3^, Jinsong Gao^1^, Xiaowei Chen^4^ & Hongbo Jia^3,9^

^1^Key Laboratory of Optical System Advanced Manufacturing Technology, Changchun Institute of Optics, Fine Mechanics and Physics, Chinese Academy of Sciences, Changchun 130033, China

^2^Graduate School, University of the Chinese Academy of Sciences, Beijing 100039, China

^3^Brain Research Instrument Innovation Center, Suzhou Institute of Biomedical Engineering and Technology, Chinese Academy of Sciences, Suzhou 215163, China

^4^Brain Research Center and State Key Laboratory of Trauma, Burns, and Combined Injury, Third Military Medical University, Chongqing 400038, China

^5^Center for Neurointelligence, Chongqing University, Chongqing 401331, China

^6^Center for Hypertension and Metabolic Diseases, Daping Hospital, Chongqing 400042, China

^7^Advanced Institute of Brain and Intelligence, Guangxi University, Nanning 530005, China

^8^Centre for Systems Neuroscience, Department of Neuroscience, Psychology and Behaviour, University of Leicester

^9^Institute of Neuroscience, Technical University Munich, 80802 Munich, Germany

Correspondence: Zhenqiao Zhou ([zhouzq@sibet.ac.cn](mailto:zhouzq@sibet.ac.cn)), Yuguo Tang ([tangyg@sibet.ac.cn](mailto:tangyg@sibet.ac.cn)), Jinsong Gao ([gaojs@ciomp.ac.cn](mailto:gaojs@ciomp.ac.cn)), Xiaowei Chen ([xiaowei_chen@tmmu.edu.cn](mailto:xiaowei_chen@tmmu.edu.cn)) or Hongbo Jia ([jiahb@sibet.ac.cn](mailto:jiahb@sibet.ac.cn))

**
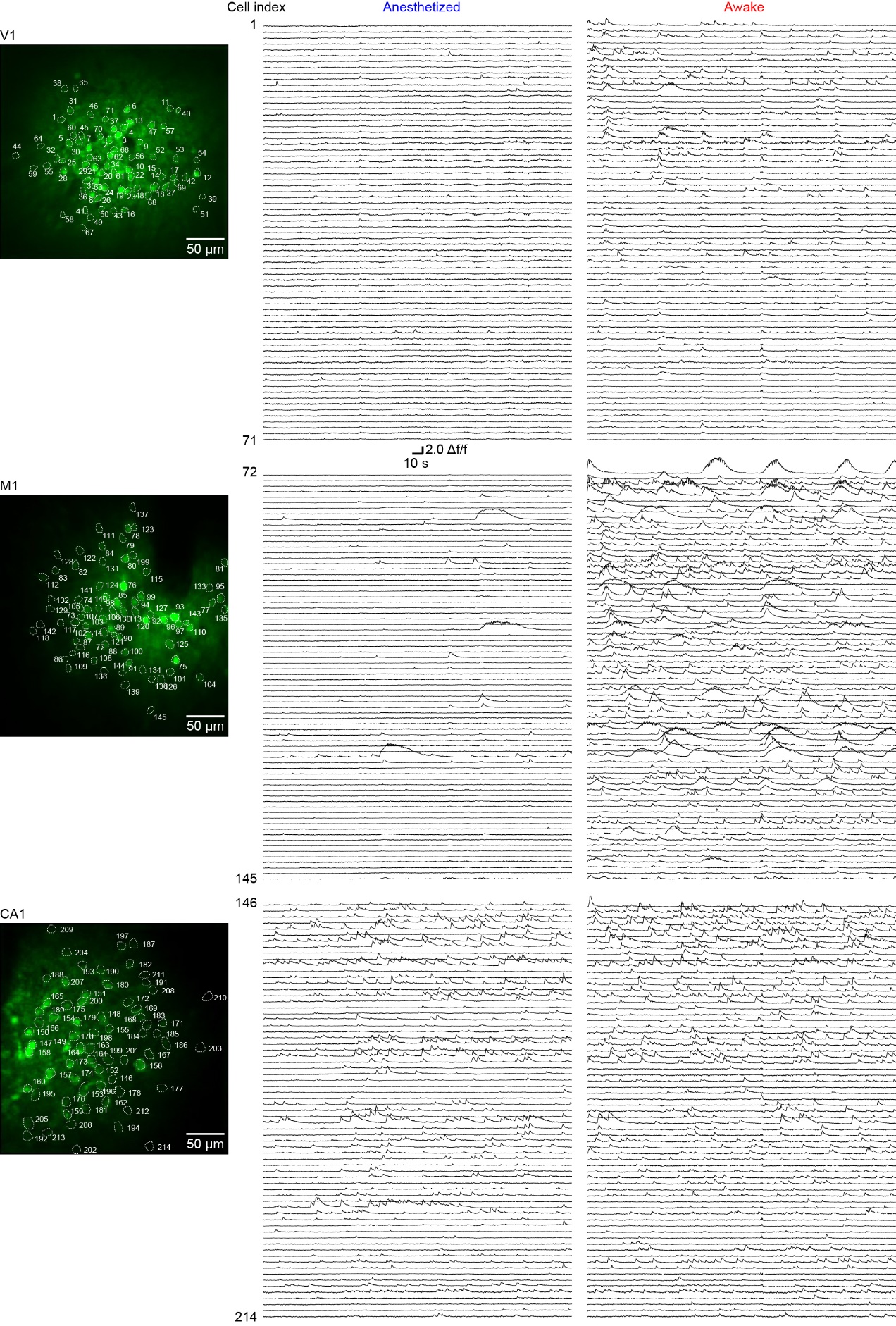
**

**Supplementary Figure 1** Complete cell-by-cell data shown for the same experiment as that in Fig. 4 d-e.


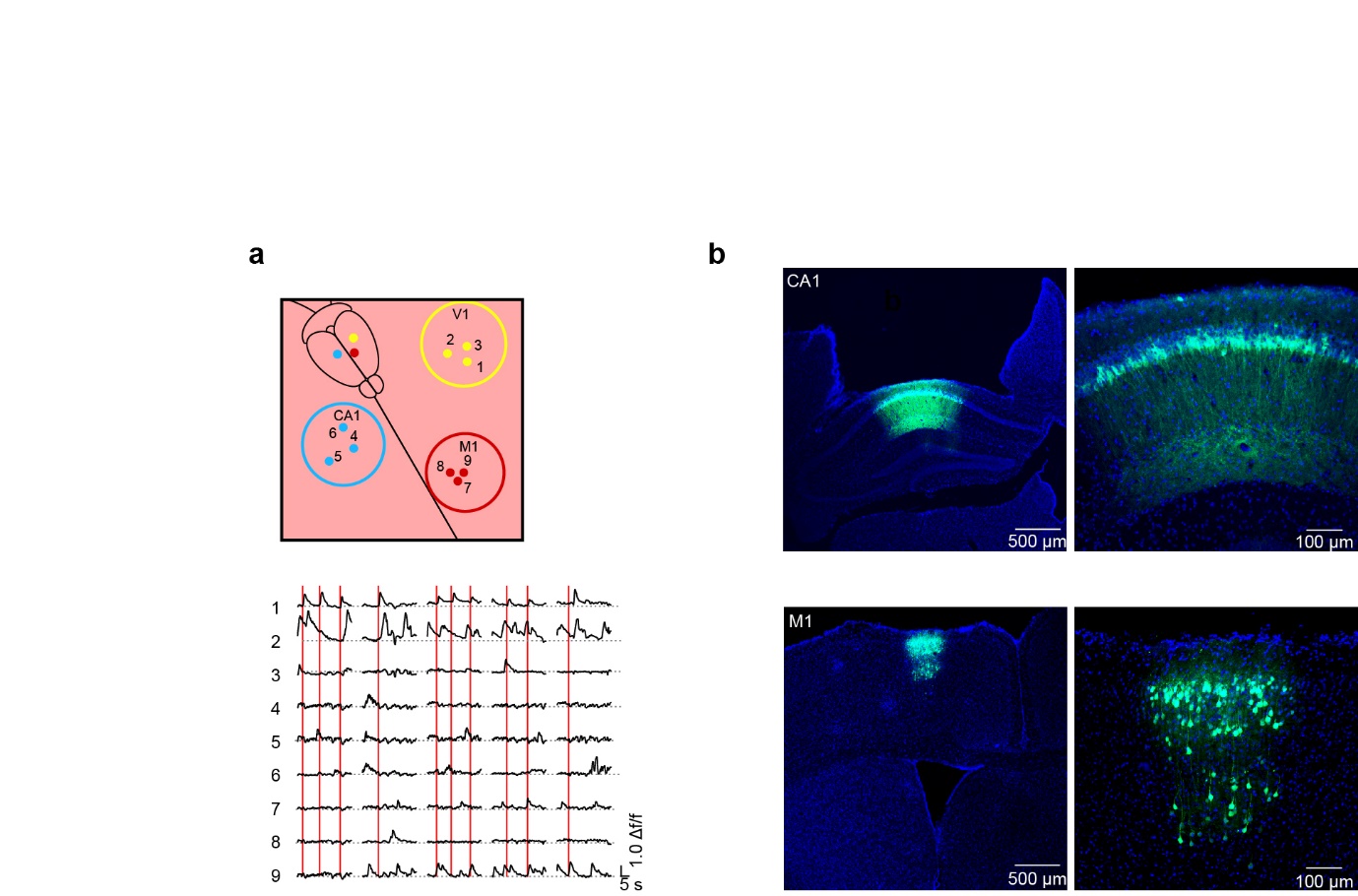


**Supplementary Figure 2.** Example of visual stimulation-related neuronal activities in V1, M1 and CA1 in awake mice. (**a**) Upper cartoon: illustration of imaging target regions with indices of selected example cells. Lower traces: Ca^2+^ signals in the 9 example cells (1-3 in V1, 4-6 in CA1, and 7-9 in M1); red vertical bars indicate LED flash stimulation events (50 ms duration each). (**b**) Postmortem brain slices from the same example animal, showing GCaMP6f-labeled neurons in the CA1 and M1 imaging regions.

**Supplementary Methods**

***Conventional resonant scanning two-photon imaging system***

An ultrafast laser (femtosecond laser, Spectra-Physics MaiTai DeepSee) was used to deliver a pulsed laser at a pulse width of ~100 fs with a repetition rate of 80 MHz at a wavelength of 920 nm and an average power of 2.3 W. The laser power fed into the scanner was modulated by a Pockels cell (PC, ConOptics model 350-120 with driver M302RM). The laser beam was expanded by a beam expander (BE, Thorlabs GBE02-B) to fill the aperture of the scanning unit. After the beam expander, the laser beam was raised to the scan box by a periscope. In the scan box, a mechanical shutter was used to stop the laser when the image acquisition was not running. The fast-axis scanner was a resonant mirror (SM1, Cambridge Technology, Model 12K CRS), and the slow-axis scanner was a galvanometric mirror (SM2, Cambridge Technology, Model 6215H). A custom-designed f-θ scan lens (SL) was used to flatten the scanning field of view in the imaging plane of the objective lens. After the scan lens, a plane mirror was used to reflex the laser to the upright microscope body that had a tri-ocular port with a built-in tube lens (TL, Olympus U-TR30IR). A dichroic mirror (DM, Semrock HC_735_LP) and a low-pass filter (Chroma ET700SP-2P8) were used to split the excitation and emission light. After passing through a collector lens (Thorlabs AC254-30-A), fluorescence photons were detected by a GaAsP detector (PMT, Hamamatsu H10770PA-40) with over 40% quantum efficiency. The back pupil of the objective lens and the photosensitive surface of the PMT were conjugated by the collector lens such that the loss of fluorescence was minimized.

A programmable data acquisition card (PXI-7851R, National Instruments) was used to control the parameters of the scanner and PMT, and a high-speed data acquisition card (PXIe-5122, National Instruments) was used to digitize the PMT output signal. The trigger bus of a PXI chassis (PXIe-1082, National Instruments) was used to synchronize the scanning trajectory and image acquisition. A customized LabVIEW software program was developed to acquire and save live streaming image data. A reconstruction algorithm was used to correct the nonlinear scanning trajectory of the 12K resonance mirror. The software features an online rolling average display function and several simple analysis functions of Ca^2+^ signal traces. The original data were saved in a TDMS stream format and could be converted to TIF or AVI format by another customized LabVIEW program.

An air-immersed objective and gradient-index (GRIN) lens, which are referred to as ‘dry objective (DO)’ and ‘mini-objective (MO)’, respectively, were used in different configurations to implement the MATRIEX technology. We offer readers our custom-made mouse head chamber (plastic, designed in *SolidWorks* and manufactured by 3D printing) and lens holders (aluminum, fine machined) to test for free, whereas the GRIN lens with the specifications shown in Table 1 can be directly purchased from <https://www.gofoton.com/>.

***Animal preparation and neuronal labeling***

Adult male C57BL/6J mice (8-10 weeks old) were used in this study. All animals were provided by the Laboratory Animal Center at the Third Military Medical University. All surgical tools and optical parts that contacted the animals were sterilized before use. All the experimental procedures were performed in accordance with institutional animal welfare guidelines, were approved by the Third Military Medical University Animal Care and Use Committee, and were similar to the procedures used in our earlier reports^1-4^.

For the GCaMP6f labeling of neuronal populations in multiple areas including both cortex and hippocampus, we performed multisite virus injections. Each C57BL/6J mouse was anesthetized with 1-1.5% isoflurane in oxygen and placed in a stereotactic head frame on a heating pad (37.5–38 °C). After we injected 100 μL lidocaine (2%) and applied ointment (Bepanthen, Germany) to the eyes, we opened the scalp with scissors and cleaned the skull using sterile cotton-tipped applicators. Then, several craniotomies (each above one target imaging area) were performed with a dental drill. We infused the virus (200 nL, AAV(2/8)-syn-GCAMP6f) through a glass micropipette with a tip diameter of 10-20 μm. The target depth of imaging in cortical regions (visual cortex and motor cortex) was approximately 150 μm beneath the cortical surface, thus we inserted the pipette tip slowly at an oblique angle of 60 degrees from vertical at an injection site -0.26 mm anteroposterior to each of the target imaging site in cortex. We then injected the virus from a vertically positioned micropipette at a depth of 1.2 mm and targeting a site in the hippocampal CA1 region. After the virus was injected at each site, the pipette was kept in place for 2 min before being retracted. The scalp incision was closed using tissue glue (3M Animal Care Products, Vetbond), and post-injection analgesics were provided for 3 days to aid recovery. All viruses were purchased from Shanghai Taitool Bioscience Corp Ltd, China. The imaging experiments were performed during the time window of 8~10 weeks post-injection.

As for surgery, the first few steps were the same as the virus injection procedure. We glued the customized chamber to the skull with UHU glue. Next, we performed craniotomies (~3 mm diameter) in each area. Then, we aspirated away the tissue over the callosum and CA1. When bleeding occurred, we applied normal artificial cerebrospinal fluid (ACSF) containing 125 mM NaCl, 4.5 mM KCl, 26 mM NaHCO_3_, 1.25 mM NaH_2_PO_4_, 2 mM CaCl_2_, 1 mM MgCl_2_ and 20 mM glucose (pH=7.4 when bubbled with 95% oxygen and 5% CO_2_) with droppers or perfusion equipment to wash the blood away.

| Reference | Lateral extent | Axial extent | Number of brain functional regions simultaneously imaged with single-cell resolution in-vivo |
| --- | --- | --- | --- |
| Tsai et al, Opt. Exp. 2015^5^ | 10 mm | ~ 500 μm | Unspecific (vascular structures were shown in-vivo, but individual cells were shown only in fixed brain slices) |
| Stirman et al, Nat. Biotech. 2016^6^ | Φ3.5mm | ~ 330 μm | 2 (2 cortical regions) |
| Sofroniew et al, Elife 2016^7^ | 5 mm | ~ 500 μm | 4 (4 cortical regions) |
| Chen et al, Elife 2016^8^ | 1.8mm | ~ 500 μm | 2 (2 cortical regions) |
| Terada et al, Nat Commun 2018^9^ | Φ6 mm | ~ 800 μm | 2 (2 cortical regions) |
| Lecoq et al, Nat. Neurosci. 2014^10^ | Unspecific | Unspecific | 2**(either two cortical regions, or a cortical region and a hippocampal region) |
| Wagner et al, Cell 2019^11^ | Unspecific | Unspecific | 2** (motor cortex & cerebellum) |
| **Our study** | Φ12 mm | >1 mm | 3 (visual cortex, motor cortex and hippocampus) |

**Supplementary Table 1.** A survey of multiregion two-photon imaging techniques. **: In these two papers the method was effectively placing two independent microscope rigs above one animal head, thus the spatial extent was unspecific, however the two imaging rigs cannot be too close to each other and one rig must be tilted in order to arrange the optic paths.

**References**

1. Wang, M. et al., Frequency selectivity of echo responses in the mouse primary auditory cortex, *Sci Rep* **8**, 49 (2018).

2. Li, R. et al., Two-Photon Functional Imaging of the Auditory Cortex in Behaving Mice: From Neural Networks to Single Spines, *Front Neural Circuits* **12**, 33 (2018).

3. Zhang, Q.C. et al., Locomotion-Related Population Cortical Ca2+ Transients in Freely Behaving Mice, *Front Neural Circuit* **11** (2017).

4. Li, J.C. et al., Primary Auditory Cortex is Required for Anticipatory Motor Response, *Cereb Cortex* **27**, 3254-3271 (2017).

5. Tsai, P.S. et al., Ultra-large field-of-view two-photon microscopy, *Opt Express* **23**, 13833-13847 (2015).

6. Stirman, J.N., Smith, I.T., Kudenov, M.W. & Smith, S.L., Wide field-of-view, multi-region, two-photon imaging of neuronal activity in the mammalian brain, *Nature biotechnology* **34**, 857-862 (2016).

7. Sofroniew, N.J., Flickinger, D., King, J. & Svoboda, K., A large field of view two-photon mesoscope with subcellular resolution for in vivo imaging, *Elife* **5** (2016).

8. Chen, J.L., Voigt, F.F., Javadzadeh, M., Krueppel, R. & Helmchen, F., Long-range population dynamics of anatomically defined neocortical networks, *Elife* **5** (2016).

9. Terada, S.I., Kobayashi, K., Ohkura, M., Nakai, J. & Matsuzaki, M., Super-wide-field two-photon imaging with a micro-optical device moving in post-objective space, *Nat Commun* **9**, 3550 (2018).

10. Lecoq, J. et al., Visualizing mammalian brain area interactions by dual-axis two-photon calcium imaging, *Nat Neurosci* **17**, 1825-1829 (2014).

11. Wagner, M.J. et al., Shared Cortex-Cerebellum Dynamics in the Execution and Learning of a Motor Task, *Cell* **177**, 669-682.e624 (2019).
